# Revisiting Human-Agent Communication: The Importance of Joint Co-construction and Understanding Mental States

**DOI:** 10.3389/fpsyg.2021.580955

**Published:** 2021-03-23

**Authors:** Stefan Kopp, Nicole Krämer

**Affiliations:** ^1^Social Cognitive Systems Group, Faculty of Technology, Bielefeld University, Bielefeld, Germany; ^2^Department of Social Psychology, Media and Communication, University of Duisburg-Essen, Duisburg, Germany

**Keywords:** human-agent interaction, conversational agents, machine learning, artificial intelligence, communication, cooperation, modeling

## Abstract

The study of human-human communication and the development of computational models for human-agent communication have diverged significantly throughout the last decade. Yet, despite frequently made claims of “super-human performance” in, e.g., speech recognition or image processing, so far, no system is able to lead a half-decent coherent conversation with a human. In this paper, we argue that we must start to re-consider the hallmarks of cooperative communication and the core capabilities that we have developed for it, and which conversational agents need to be equipped with: incremental joint co-construction and mentalizing. We base our argument on a vast body of work on human-human communication and its psychological processes that we reason to be relevant and necessary to take into account when modeling human-agent communication. We contrast those with current conceptualizations of human-agent interaction and formulate suggestions for the development of future systems.

## Introduction

Building computer systems that are able to converse autonomously and coherently with a human is a long-standing goal of Artificial Intelligence and Human-Computer Interaction (going back, at least, to the seminal *Eliza* system presented by Weizenbaum, 1966). The history was marked by ups and downs, and there have been numerous coordinated research endeavors in particular in the last two decades directed to the realization of so-called “conversational agents” (Cassell et al., 2000) or “conversational artificial intelligence (AI)” (Ram et al., 2017). Still, today, conversational agents have not fulfilled the common expectation that, by trying to advance and combine abilities for verbal and nonverbal communication, the interaction with computers and technology will be facilitated and rendered more intuitive. So far, no system is able to lead a half-decent coherent and engaging conversation with a human user. Even the voice-driven applications people use in everyday life – in-car navigation, smart speakers at home, or personal assistants on smartphones – merely enable only task-specific “dialogs” that consist of spoken instructions or questions by the user and direct responses by the system that often do not meet user expectations (Luger and Sellen, 2016). This is seemingly in contrast to the considerable progress in recent years with the advent of powerful machine learning techniques that have leaped language processing to a new level. Yet, these approaches rely on large datasets from which they process isolated snippets of spoken language use, and this is what they enable – the processing of single spoken inputs, or the generation of answers to individual requests.

Given the remarkably large discrepancies in effectivity and flexibility between human-agent communication and natural human-human communication and conversation, we argue that we continuously need to question the directions in which the field is heading. Even though human communication is not error-free either, it turns out to be astonishingly resilient, robust, and efficient in establishing sufficient coordination between interlocutors. To achieve this, humans use verbal and nonverbal means to make themselves understood, and to mutually know they do at the same time. But, considering frequently made claims of “super-human performance” in, e.g., speech recognition or image processing, why are technical systems still falling short of this? One goal of the present paper is to (re-)emphasize the hallmarks of human communication and its complexity, and to argue that we should not lose sight of these hallmarks when deriving requirements for human-agent-interaction (HAI). We hold that it is in particular some of the core capabilities that humans have evolved for natural conversational interactions that are still largely missing in current technical approaches and are, unfortunately, increasingly getting out of focus.

We do not suggest that conversational agents should be built to emulate all attributes of human-human interaction (HHI; let alone and intelligence) up to complex intersubjective meaning or emotional-relational qualities like empathy, affiliation, or friendship. HAI and HHI should be considered as different kinds of encounters. However, we do want to highlight basic prerequisites and abilities that, as we argue, are indispensable for conversational agents to eventually enable more complex, robust, and effective dialogs with human users, and hence to become more useful and acceptable systems. That is, although HAI will for a long time be bound to differ qualitatively from HHI in form and function, conversational agents need to be equipped with core abilities to make HAI similarly efficient, robust, and powerful as HHI. We base our argument on a vast body of work on models and findings on human-human communication and its psychological processes that we deem also to be relevant and necessary to take into account when modeling human-agent communication. This includes crucial insights from Cognitive Science and Psychology that contemporary AI and engineering has, for some part deliberately, started to ignore. We do not criticize or address machine learning and natural language processing (NLP) experts here, though. Their methods have brought about numerous technological advancements in various realms such as spoken-language based interfaces and text processing. This paper is directed more at the community of researchers building socially intelligent agents or robots, who consider which route to take best to improve future HAI with hallmarks of situated, multimodal, and flexible conversation, and who need to investigate to what extent contemporary AI methods can help achieve this goal.

Our starting point is to acknowledge and re-iterate the fundamental importance of *cooperation* as the basic prerequisite for, and distinct characteristic of human dialogical communication. As early as 1975, Grice pointed out that cooperation is at the heart of human-human communication. More current conceptualizations stress the evolutionary background of humankind’s urge to cooperate and underline the importance of our corresponding “psychological infrastructure of shared intentionality” for our everyday communication (Tomasello, 2008). We posit that cooperative communication rests on two crucial mechanisms that allow humans to achieve mutual understanding in a dialog, and consequently that have to be incorporated more adequately in conversational agents or robots: the primacy of *joint co-construction* as the stepwise construction of a joint activity and the primacy of *mentalizing* as the ability to perceive, understand, and predict an interlocutor’s relevant mental states.

### Primacy of Joint Co-construction

Ethologists, psychologists, and communication scholars have aptly described the fact that every person individually constructs how she views the world. It is *via* communication and the observation of the other’s behavior that an interlocutor tries to understand the other person’s mental states (i.e., entirety of all beliefs, intentions, goals, attitudes, feelings, etc.), but one can never be certain whether (a) the interpretation of the other’s mind is complete or correct and (b) own communication and interpersonal signals are interpreted by the other in the way they were intended to Watzlawick et al. (1967); Burgoon and Bacue (2003). Humans can overcome these problems by what we term “joint co-construction,” through stepwise testing of increasingly complex hypotheses about the other’s mental state and how it can be changed toward the goal of the interaction, and by grounding this in incremental and highly responsive dialog moves. This interactive “*think enough, speak and listen more*” approach enables interaction partners to iteratively co-construct their interaction and their mental states at the same time. Contemporary conversational AI, in contrast, follows the more opposite approach of “*think (and predict) more, speak less*,” based on the assumption that all necessary reasoning for a suitable, self-contained communicative response can be done on-the-spot and based on a sufficiently large set of training data.

### Primacy of Mentalizing

A decisive question is what a social cooperative agent needs to perceive and know about its interaction partner, the interaction, and itself in order to be able to produce behavioral strategies that are needed to lend mutual support and to truly cooperate in co-constructing a dialog. Contemporary machine learning-based systems are reminiscent of behaviorist approaches by trying to extract patterns of communicative conduct from large amounts of surface-level data and using them based on correlations with input features at the same level. We propose that a holistic and more cognitivist “mentalizing” approach is needed that encompasses an ability to perceive, interpret, and understand the interlocutor’s relevant mental states as a precondition for a smooth, coherent dialog. Relevance, here, refers to the causal role of such mental states for the individual choice of interactive behavior, and they exceed the level of discrete dialog or belief states commonly considered. An important prerequisite for achieving this is to be able to reconstruct the construction the (human) interaction partner does in his/her mind, i.e., to differentiate and predict mental perspectives of the interaction partner(s).

So far, there has been no approach in the realm of conversational agents or social robotics that would adequately implement these prerequisites for a dialogical interaction that is likely to yield mutual understanding. This paper is – to the best of our knowledge – the first attempt to look at the basic human abilities of joint co-construction and mentalizing from an interdisciplinary perspective including psychology and computer science, and to suggest how they can be integrated in a holistic approach to improve dialog abilities in HAI. This includes first ideas about how cognitive AI, which considers the nature and processes of human cognitive abilities (cf. Zhu et al., 2020), can be complemented by data-based machine learning approaches but not be rendered dispensable by them.

The paper, therefore, combines a review of relevant literature and then, in a pleading to not jettison cognitive approaches, we advocate a combination. In the following section How Humans Communicate and Cooperate in Dialog, we will review a wide range of theories and models which illustrate the complexity of human-human communication and underline these two primacies. In section Computational Models of Human-Agent Communication, this is contrasted with a discussion of current conceptualizations of HAI, both from cognitive AI and machine learning. We then formulate suggestions for the development of future systems that specifically take into account the importance of incremental co-construction and mentalizing. In the concluding section, we discuss how this, in turn, may also inform and inspire extensions of contemporary modern data-based approaches to conversational agents.

## How Humans Communicate and Cooperate in Dialog

“*It is the nature of the human condition that, try as we may, we cannot enter into the reality of another individual’s experiences, thoughts, or feelings. Imprisoned as we are within our own bodies, the fallible process of communication is the primary agent currently available for crossing the psychological expanse between two or more individuals*”

(Burgoon and Bacue, 2003, p. 179).

The quotation aptly describes human communication as a wonderful means to connect people – still being the only way to access others’ feelings, thoughts, and experiences. Although seemingly effortless and simple, our capacity to communicate is a very complex process which regularly becomes apparent when we encounter disruptions such as misunderstandings or unintended effects. The complexity also becomes evident when trying to emulate abilities for communication in artificial entities, which is why all existing systems need to start out with simplifying assumptions about human communication. The crucial question, then, is which assumptions are needed, warranted, or what their implications are. Or, the other way around, the question is which mechanisms or abilities are crucial for communication and thus ultimately indispensable also for artificial systems. The goal of the present section is to underline the manifold abilities needed to be able to communicate with others in the way humans do. We thereby draw on theories from social and communication psychology, ethology, linguistics, and cognitive science in order to understand the most important accomplishments of human cognition that lay the ground for communication. We would like to stress that – although it will not be discussed at the core of this paper – sociolinguistic approaches (Dickerson et al., 2013; Broz et al., 2014; Rollet and Clavel, 2020) play an important role for the improvement of current conversational systems.

We start out with an account of what makes human communication so intricate and difficult to model. One main reason for this is the fact that messages are not necessarily understood in the way they were planned to be. For example, various currently implemented models of human-technology interaction implicitly assume that the information that is sent by the technology is perceived and interpreted by the user in the intended way. However, nowadays, it is widely acknowledged that neither verbal nor nonverbal interaction is best viewed as a one-to-one transmission of meaning from sender to receiver. Especially representatives of constructivist assumptions or general systems theory (Watzlawick et al., 1967) assume that meaning is not fixed, encoded into a signal, transmitted and then decoded, but that it is constructed by the receiver and depends heavily on his/her perception of situation and context. And this evolving, subjective interpretation of the receiver needs to be monitored by the speaker and compared to her intended meaning. Dialog modeling, therefore, can only be successful when the fact that individual interpretations occur are taken into account properly.

Consequently, human communication must be seen as co-determined by the receiver’s abilities, attributes, and current state. Especially, communication models originating from systems theory stress that the receiver’s current “structure” affects the decoding of a message (Watzlawick et al., 1967; Maturana and Varela, 1984). Instead of decoding and “understanding” the message in exactly the way it was intended, the human receiver constructs and interprets the message. The elemental conclusion that can be derived is of course that this renders human communication difficult because it entails that the sender has neither control nor direct knowledge of how the receiver will decode and interpret the message. The human sender thus will have to estimate the effects of her utterances based on knowledge about the receiver (ranging from basic knowledge of human nature to specific knowledge about the person she might be familiar with, see mentalizing ability below), the situational context they both share, and signals received back from her. That is, within the ongoing conversation, the cues emitted by the interlocutor in form of her utterances and nonverbal reactions are used for hypotheses building about whether one’s own utterance was understood as intended or not (see incremental co-construction as described in greater detail below).

### Humans’ Core Abilities to Cooperate as a Basis for Communication

It has been argued that the most basic capability of humans that enables all sorts of social interactions is their ability to cooperate, not only with regard to physical interaction but also with regard to communication and dialog interaction. Probably most influentially, Tomasello (2010) has described and demonstrated humans’ cooperativity. He stresses humans’ unique “psychological infrastructure of shared intentionality” which denotes the ability to develop joint goals and to support each other mutually, for example, *via* the sharing of information. Importantly, for humans, the sharing in itself is a rewarding activity that leads to hedonic joy and pleasure (satisfying a “social motive”). Tomasello (2010) aptly illustrates this with the example of an 18-month-old kid who excitedly tries to inform her grandfather about the fact that her father has just set up the Christmas tree. Here, the simple fact to be able to share this information and to jointly admire the Christmas tree is rewarding. No other species has similar abilities, which is why Tomasello (2014) states that humans are “ultra-social animals.” This ultra-sociality is grounded in both cognitive and motivational mechanisms, that is, on the one hand, humans have an innate urge to cooperate and, on the other hand, they have cognitive abilities which are optimized to support joint activities. To collaborate and to help others seems to be intrinsically rewarding: 14-month-old infants were observed to help adults with all kinds of tasks without a concrete reward (Warneken and Tomasello, 2007) and provide others with information that they need (Liszkowski et al., 2008). In parallel, scholars from other disciplines have described the social nature of human actions, for example, in sociology (Enfield, 2013) or philosophy (Bratman, 1992). For example, Bratman (1992) describes that humans are able to manage shared cooperative activities because of a trio of features: mutual responsiveness, commitment to the shared activity, and commitment to mutual support.

#### Cooperation in Dialog and Communication

The fact that cooperation is at the heart of human interactions is clearly visible with regard to language and interaction. The fabric of dialog itself is cooperative or else no meaningful interaction would be possible (Tomasello, 2010). Simple communicative acts, such as pointing to a bike, require not only a common ground in the sense of a common context in order to be meaningful, but they also require the knowledge and commitment that the other person wants to notify something that is relevant to her. At the same time, language and communication are used in order to organize and structure cooperation. *Via* communication, interaction partners build a mutual understanding on when cooperation begins and ends. In this line, Gräfenhain et al. (2009) demonstrate that even 3-year-olds “take leave” through either implicit or explicit communication when they need to break away from a joined commitment. There is also communication and awareness about roles and the tasks of the other. While chimpanzees cannot easily switch roles in a collaborative activity, children already look for and know what to do from having observed the partner (Fletcher et al., 2012). This is in line with well-established assumptions about the fundamentally cooperative nature of language and dialog (Grice, 1975). Allwood et al. (2000) define cooperative behavior in dialog by four attributes: cooperative interaction partners (i) take the cognitive states of the interlocutor into account; (ii) follow a joint goal; (iii) support the other in achieving his/her goal; and (iv) trust each other mutually to adhere to the former aspects (i)–(iii).

#### Incremental Co-construction for Cooperation in Dialog and Communication

Language use has been described as inherently collaborative in giving each other moment-by-moment feedback and evidence of understanding (Brennan, 1991, 2005). This process can only proceed incrementally and co-constructed by the sender and the receiver, who are working in a step-by-step manner to achieve and provide each other with sufficient evidence of sufficient mutual understanding. Each and every communicative act or utterance in a dialog thus must be seen to be embedded in and to derive from the immediate communicative and mental contexts. This includes prior adaptation to the interlocutor during the planning of one’s own contributions, as well as a posterior and online adaptation based on the recipient’s feedback signals or displays. Both mechanisms represent two sides of the same coin – that interlocutors are able to take their addressee’s perspective and adapt to it swiftly and cooperatively.

Levinson (2006) points out empirically-observable cooperative practices in everyday communication – turn-taking, sequence templates, and repair among them – and identifies a set of underlying mechanisms as the human “interaction engine” such as the attribution of relative beliefs, higher-order mental states, or Gricean communicative intentions to others. Dingemanse et al. (2015) nicely exemplify the universality of these practices by demonstrating that in 12 different languages, similar repair mechanisms can be found (one per every 1.4 min of conversation) which are largely cooperative: people follow the common principle of specificity, i.e., choosing the most specific repair initiator possible. While the egocentric, non-specific strategy would be to choose the simplest form possible (e.g., “huh”) leaving the work to the interaction partner, people select a behavior that rather minimizes collaborative effort. Brennan and Hanna (2009, p. 274) also explicitly define dialog as a cooperative act: “*Spoken dialog is a form of joint action in which interacting individuals coordinate their behavior and processing moment by moment and adapt their linguistic choices and nonverbal behavior to each other*.” The coordination happens *via* communication itself, in an activity that can be compared to a negotiation (Rapaport, 2003) or “hypothesis testing” (Brennan, 1998).

#### Mentalizing in Dialog and Communication

As described above, a crucial source for the complexity of human communication is that recipients do not simple decode messages but construct them against the background of their prior experiences as well as mental and affective states. In order for human communication to work smoothly, a basic understanding of the interlocutor’s mental states is important. This understanding can be reached by forming a representation of others’ beliefs and attitudes and updating them given new information (building on a general ability for “mentalizing,” Frith and Frith, 2006). Brennan et al. (2010) also describe cognitive processes such as mirroring and mentalizing that provide the basis of coordination during communication by allowing speakers and listeners to adapt flexibly to the perspective of a conversational partner. Even theories that posit an “egocentric tendency” in (early) communication-related processing (Keysar et al., 2000) acknowledge that conversational partners possess and employ an ability for perspective-taking based on partner-specific information. Along similar lines, Teufel et al. (2010) develop a model of social perception based on cognitive neuroscience and conclude that the mere observation of social signals of others (e.g., physical characteristics) leads to “perceptual mentalizing,” i.e., automatically inferring and attributing mental states to them.

**Figure 1:**
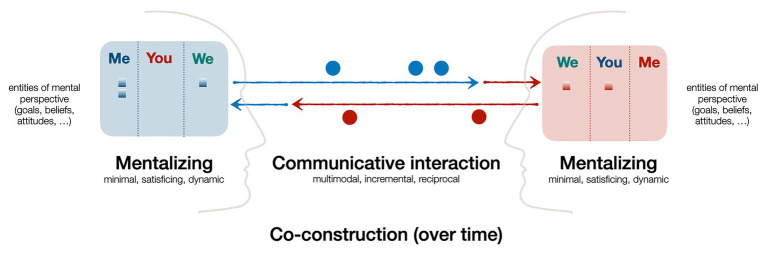
Schematic overview of the role and interplay of mentalizing and incremental co-construction in cooperative communicative interaction (see text for explanation).

In summary, communication inherently requires, and yields, cooperation, and this rests on incremental co-construction and mentalizing. It can even be stated that these two are intertwined and two sides of the same coin of socially cooperative communication (see Figure 1): mentalizing refers to one’s ability to perceive, distinguish, and predict the interlocutors’ mental states and perspectives. This underlies and drives forward the process of communication, whose main goal is to achieve a sufficient mutual (“we”-) understanding of a particular issue or topic. Doing this cooperatively means to adapt one’s communicative actions to the interlocutor, both prior to saying something and then continuously based on the interlocutor’s feedback signals or other response. In result, as this plays out by and within all interlocutors in parallel, both the overt communicative interaction as well as the covert mutual understanding and deepening of common knowledge are incrementally co-constructed.

### Theoretical Models of Human Communication

Different disciplines have developed models to explain the fact that humans can communicate effectively and successfully. These models all draw on the fact that sender and receiver have fundamental similarities since they share human processing with regard to needs, thoughts, emotions, etc. The assumption is that this enables the speaker to design messages to be appropriate to what she/he perceives to be the knowledge of the recipient (audience design hypothesis; Clark, 1992; Fussell and Krauss, 1992; see 7). In psychology, models on perspective taking (Krauss and Fussell, 1991), common ground (Clark, 1992), imputing one’s own knowledge in others (Nickerson, 1999, 2016) have been proposed. Additionally, the basic ability of a “theory of mind” (Premack and Woodruff, 1978) has been described which might be understood as a meta-theory to unify the different approaches (Krämer, 2008).

#### Perspective-Taking

Krauss and Fussell (1991) describe the process of perspective-taking in communication from a social psychological point of view and state that the failure to take other’s perspective can be the basis for misunderstandings and dispute. A prerequisite for successful communication is that the message is tailored to the knowledge of the recipient. Empirical evidence shows that the accuracy of people’s assessments of others’ knowledge is fairly high but that people, on the other hand, seem to be biased in the direction of their own knowledge (see also Nickerson, 1999, see below). Krauss and Fussell (1991) conclude that people’s assumptions about other’s knowledge are necessarily tentative and best thought of as hypotheses that need to be evaluated and modified over time. Similarly but with a focus on nonverbal reciprocity, Burgoon and White (1997, p. 282) stress the importance of this mutual “online” adaptation and joint construction of the other’s knowledge in their “interaction adaptation theory”: “All message production, and especially that in interpersonal conversation, implicitly begins with an alignment towards the message recipient and the predisposition to calibrate one’s messages to the characteristics of the target (as well as the topic, occasion, and setting).” Therefore, the theory entails both: mentalizing and incremental co-construction.

#### Managing Common Ground

Similarly, Clark (1992, p. 93) describes common ground as the joint basis for communication: “Two people’s common ground is, in effect, the sum of their mutual, common, or joint knowledge, beliefs, and suppositions.” He assumes common ground to be a sine qua non for everything humans do with others: to coordinate and communicate with others, humans have to appeal to their current common ground. This implies that in case there is no common ground no communication or understanding, respectively, would take place: to illustrate this, he aptly quotes Ludwig Wittgenstein who, in his philosophical investigations, stated: “If a lion could talk, we could not understand him.” Therefore, it can be assumed that there should be an initial common ground in each conversation that can be broadened during the interaction. The most obvious starting point in terms of communal common ground is human nature. As an example, he points out that if a sound is audible to someone, he/she will assume that it is also audible to the other, that people take the same facts of biology for granted, that everyone assumes certain social facts (people use language, live in groups, and have names). Similarly to the approach by Krauss and Fussell (1991; see above) it is further assumed that the actual conversation can be used for preventing discrepancies. Humans have verbal and nonverbal strategies to discover and repair situations when the mutual knowledge is misinterpreted. “Contributors present signals to respondents, and then contributors and respondents work together to reach the mutual belief that the signals have been understood well enough for current purposes” (Clark, 1992, p. 252). This process is specified in Clark and Schaefer (1989) who stated that positive evidence for understanding generally arrives in five categories of increasing strength: “continued attention” (i.e., without any repair initiation), “initiation of the relevant next contribution,” explicit “acknowledgment” (possibly *via* back channels or multimodal signals), as well as “demonstration” and “display,” referring to (partial) paraphrase or cooperative completion and verbatim repetition, respectively. In sum, the theory also includes both, mentalizing (here termed communal common ground) and incremental co-construction (by slowly increasing the common ground *via* communication).

#### Imputing One’s Knowledge to Others

Drawing on concepts like perspective taking (Krauss and Fussell, 1991), common ground (Clark, 1992), or emphatic accuracy (Ickes, 1993; i.e., ability to accurately infer the specific content of another person’s thought and feelings). Nickerson (1999, p. 737) forms a model of how humans build beliefs on the knowledge of their interlocutors that he sees as an important prerequisite for communication: “To communicate effectively, people must have a reasonably accurate idea about what specific other people know. An obvious starting point for building a model of what another knows is what oneself knows, or think one knows.” He thus assumes the ability to impute one’s own knowledge (including beliefs, opinions, suppositions, and attitudes) to others to be vital for human-human communication. The model he proposes is tailored to the case that one needs a model of what a specific individual knows (e.g., when directly communicating with him/her). If nothing about the specific individual is known, a model of one’s own knowledge and considering potential unusual aspects of one’s own knowledge, as well as any information on the specific individual may be the best one can do. In this process-oriented model, the mentalizing is at the start of the model when the first estimation of other people’s knowledge is made. Incremental co-construction can happen in the following when trying to validate the assumption.

#### Theory of Mind

“Theory of mind (ToM)” is the ability to understand other entities as intentional agents, whose behavior is influenced by hidden mental states like beliefs, goals, feelings, etc., and the knowledge that other humans wish, feel, know, or believe something (Premack and Woodruff, 1978; Premack and Premack, 1995). This entails a direct understanding of what other people know or might feel in a specific situation. In recent years, ToM has been discussed as a basic prerequisite for HHI and various terms have been established: mentalizing (Frith and Frith, 2003), mindreading (Baron-Cohen, 1995), and intentional stance (Dennett, 1987) all basically refer to the same ability that is seen as crucial for all aspects of our everyday social life and our natural way of understanding the social environment: in this line, Dan Sperber stated that “attribution of mental states is to humans as echolocation is to the bat” (Baron-Cohen, 1995, p. 4). Also, Tooby and Cosmides (1995) stress the function and innateness of the ability: “We are ‘mindreaders’ by nature, building interpretations of the mental events of others and feeling our constructions as sharply as the physical objects we touch. Humans evolved this ability because, as members of an intensely social, cooperative, and competitive species, our ancestors’ lives depended on how well they could infer what was on one another’s minds” (Tooby and Cosmides, 1995, p. 13). Indeed, ToM has been discussed as a prerequisite for communication between human interactants: although “mindreading” does of course not allow for a 100% correct prediction of mental states, it provides a general orientation on other people’s processes and a prediction of the effects of communication. Baron-Cohen (1995, p. 27) thus sums up: “A …reason why mindreading is useful, and thus why it may have evolved, is the way in which it allows us to make sense of communication.” Unlike the other theories and models, ToM heavily relies on the aspect of mentalizing and does not refer to incremental co-construction.

In summary, the different perspectives of common ground, perspective taking, imputing one’s knowledge or ToM show major similarities with regard to the assumption that humans possess direct but implicit knowledge of other humans to form a starting point for mutual comprehension. This is in line with what we refer to as “mentalizing” in this paper. Originating in this, and together with the innate ability and willingness to cooperate with each other toward joint communicative success, human dialog emerges as the reciprocal cooperative attempt to establish mutual knowledge by means of grounding processes – in the sense of incremental co-construction. The findings and insights that we have discussed here underscore that the principles of mentalizing and incremental co-construction are indispensable for this and must also be taken into account when trying to model dialog with technical systems.

Note that we advocate the inclusion of these principles not only for the mere sake of maximizing naturalness or smoothness of human-agent communication. Rather, we suggest that these mechanisms are fundamental to the basic functioning and efficacy of a conversational interaction overall, and even more so for attempts to model it between humans and technical artifacts with their (still present) limitations in recognizing or synthesizing human verbal or nonverbal communication. The basic argument is that communication is bound to be limited when the human user cannot build on his/her mentalizing ability when, e.g., trying to address a machine. Also, the machine is not enabled to engage in incremental co-construction when it does not know where to start from. One fundamental question is here whether – even if HAIs will be optimized by increasing agent communication abilities – human-agent communication and human-human communication will always differ due to the fact that artificial agents are perceived and treated differently compared to fellow humans. On a higher level (e.g., with regard to relationship building) there will (and most probably should) always be differences. However, on a basic level of understanding each other cognitively or affectively, it can be expected based on findings related to media equation assumptions (Reeves and Nass, 1996; Nass and Moon, 2000; Krämer, 2005) that humans display “normal” behavior in the sense of deeply-rooted social and communicative actions toward artificial entities.

## Computational Models of Human-Agent Communication

Enabling human-like communication with artificial agents has been a long-standing goal in AI and Human-Computer Interaction, dating back to the early suggestion of taking it as a hallmark and measure of (machine) intelligence in the Turing test. Corresponding abilities have been investigated for spoken dialog systems (McTear, 2002), embodied conversational agents (Cassell et al., 2000) and sociable robots (Fong et al., 2003) that are embodied and can also produce nonverbal behavior (e.g., gestures, facial displays, and gaze). This trend has been accelerated by a large-scale application of voice-based interfaces, smart speakers, or personal assistants such as Amazon Alexa, Google Echo, Microsoft Cortana, or Apple Siri. Recently, there is a burgeoning interest in social chatbots (“socialbots”) which are expected to not only respond to users’ specific questions or commands in natural language (spoken or typed), but also to establish a connection by tracking the user’s emotional state (Li et al., 2018) and satisfying users’ social needs for communication, affection, entertainment, or belonging (Shum et al., 2018). In this section, we will review approaches that have been adopted – classically and recently – for realizing human-agent communication, before discussing in Discussion and Future Research Directions section, how this abides by (and deviates from) the underlying principles of human communication and pointing out directions that future research may explore. Thereby, we predominantly address short-term interactions as we do not explicitly refer to modeling memory and other aspects necessary for longitudinal interactions. Still, we consider longitudinal interactions to be eventually crucially important and the ultimate goal of human-agent communication. What is suggested here will serve as a prerequisite for any further steps in this direction.

In general, technical approaches rely on the classical “fixed code model” of communication dating back to Shannon and Weaver. That is, they are built to support a repertoire of communicative signals identified and modeled during design and implementation of the system. Specialized components are developed or trained to process these signals as input or to generate them as output, e.g., for recognizing speech, interpreting facial expressions or hand gestures, multimodal fusion, or synthesizing multimodal behavior. Such components are connected in processing pipelines structured according to an overall architectural layout. This modularization and decomposition of dialog processing is a logical approach given the daunting complexity of modeling communication. Recent statistical dialog systems (Ultes et al., 2017), for instance, break the pipeline down into a *semantic decoder* (transforms text to a semantic representation), a *belief tracker* (maintains the internal dialog state representation), a *topic tracker* (identifies the current dialog domain), a *policy* (maps the belief state to a system dialog act), and a *language generator* (transforms the dialog act into text). A range of methods have been developed for these individual tasks; see, e.g., Jurafsky and Martin (2008). State tracking and the dialog policy constitute the so-called dialog manager and several different approaches have been proposed to this problem. While early approaches were rule-based (using flowcharts or finite state machines), the second generation dialog systems were based on probabilistic and statistical methods. For example, partially observable Markov decision process (POMDP) was used to formalize dialog behavior as rational decision-making under uncertainty (Young et al., 2013), which also allowed for optimizing the dialog policy by means of reinforcement learning. In recent years, a new generation of dialog systems built around deep (machine) learning has emerged, which still adopt the structure of the statistical dialog systems, but apply neural network models in each module. Two general trends can, thereby, be observed: first, moving from structured-symbolic approaches with explicit features (grammars, rules, templates, finite state machines, etc.) toward implicit mappings extracted from very large datasets by statistical or neural learning techniques (Chen et al., 2017). Second, and building on the first trend, an increasing number of approaches aim to resolve modular processing structures and layers by trying to learn a direct “end-to-end” mapping from input forms to output forms (Serban et al., 2016).

### Data-Based Conversational AI

Current ML-based approaches are trained on spoken language data in order to statistically model a mapping from natural language or text input to output. They can be subsumed under the term data-based “conversational AI.” Their increasing availability and popularity have greatly promoted the development and deployment of spoken language-based HAI. Almost all current dialog systems (and certainly the ones commercially used) are based on such techniques. They are usually task-oriented and geared to support specific forms of dialog in a given domain or a small number of domains (Gašić et al., 2015; Serban et al., 2017). There are two main approaches (cf. Huang et al., 2020): on the one hand, *retrieval-based* systems use a large repository of conversations from which, for any user message, the most appropriate response is retrieved and then outputted to the user. This casts response generation into a search problem (Ji et al., 2014) and much work has attempted to improve the quality of the dialogs achievable in this way, e.g., by including topical or contextual information in this retrieval problem (Wu et al., 2018). On the other hand, *generation-based* approaches try to construct new responses for new messages by optimizing for certain criteria. A majority of these methods use deep learning techniques to model a sequence-to-sequence mapping. This approach is robust and generic, but often leads to responses that may be off-context, uninformative, or vague (Vinyals and Le, 2015). Much work is thus being directed to fine-tune these response generation methods by taking more general aspects such as local context, personality, emotions, or attention into account.

Another goal has been to develop methods for building generic or open-domain systems that do not require predefined features or state spaces (Serban et al., 2016). For example, conversational systems usually need a specific approach for tracking the state of the dialog. This includes finding an appropriate representation for the relevant features that account for dependencies in conversational behavior. Likewise, the dialog policy is optimized for certain dialog or task measures (e.g., semantic coherence, information flow, and group coordination). Open-domain dialog systems aim to overcome these limitations and are increasingly tackled due to the availability of large amounts of conversational data and progress on neural approaches. As pointed out by Huang et al. (2020), current attempts aim to address the challenges of understanding the user (not only the content of words), producing consistent behavior, and ensuring long-term user engagement. In particular, ensuring the coherence of dialog responses and maintaining long-range dependencies as needed for, e.g., grammatical, semantic, discourse, or pragmatic consistency over longer outputs and multi-turn dialog segments is a subject of ongoing research. Modern approaches aim to account for this in a bottom-up fashion, by increasing the amount of training data and by introducing special means of capturing contextual information in hierarchical models or with hidden (latent) variables (Serban et al., 2017; Tao et al., 2019). In the realm of language models, longer-range dependencies are captured by integrating special “attention” mechanisms into sequence-to-sequence processing. The most recent trend is the non-recurrent neural network “Transformers” architecture. The largest such model developed so far is the GPT-3 model presented by OpenAI (Brown et al., 2020). This language model was shown to be able to complete different kinds of texts, to translate between language, to correct language errors, or to solve simple arithmetic problems. Interestingly, GPT-3 also performs quite well on semantic and discourse reasoning tasks, such as resolving pronouns or predicting final words of sentences. In light of the impressive achievements of GPT-3, an interesting discussion has been unfolding regarding the ability of such language models to present a “general AI,” and whether the often attested lack of meaning or understanding of such models that mainly operate at the level of forms (Bender and Koller, 2020) is even relevant.^1^ Indeed, the fascinating performance in many tasks demonstrates that many patterns underlying semantically coherent language use can be extracted and synthesized when scaling up the models and the data. At the same time, tests have shown that GPT-3 does produce unexpected or inconsistent behavior when answering questions or conducting dialog. While this has been attributed to a lack of real-world grounding of understanding in text-based models,^2^ we point out that what is also completely missing in such language models is the level of social intelligence underlying conversational interactions (cf. How Humans Communicate and Cooperate in Dialog section).

In sum, many approaches in conversational AI have purposefully abstracted away from the underlying processes of human communication and dialog, while focusing on optimizing local, surface-level behavior (for the most part, single responses) based on large amounts of training data and integrating linguistic, task domain, and world knowledge. This has led to improved system abilities for robust spoken-language dialogs in structured tasks and within confined domains. At the same time, however, it is increasingly apparent that purely behavior-based approaches (mapping word sequences onto word sequences) are insufficient and need to be augmented with additional information about the interaction context or the speakers. There is promising work on open-domain dialog systems and social conversational agents, which, however, still has to solve many challenges in order to provide models that can reliably deal with the ambiguities, vagueness, or subtleties of human communication. For instance, in natural communication, “speaker meaning” (what the speaker *intends* to communicate) often is not directly indicated through the “literal meaning” of communicative acts (Grice, 1975), but is rather implied by communicative acts that involve multiple intentions and are produced by individuals with private, subjective beliefs, and individual conceptions of language and the world (Sperber and Wilson, 1986). Understanding and generating natural language thus requires computational models that bring to bear not only extensive linguistic knowledge (from phonetic to morphological, lexical, syntactic, semantic, and pragmatic levels) but also deep knowledge about the interaction partner, the current or previous discourses, and the shared situational context. We argue that such models cannot be learned *a priori* from large amounts of data alone, but also need to be incrementally co-constructed by interlocutors on the spot.

### Agent-Based Approaches to Conversation

Our current impression is that many contemporary approaches have deviated from original modeling attempts, which have started from the decisive goal to model human-like abilities for conversation, not just the behavioral regularities and patterns in language-based encounters. Early work on conversational agents has taken an interaction-oriented approach to dialog that adopts the view that conversation is a joint activity (Clark, 1992), in which interlocutors “coordinate” the information sources underlying ostensive-inferential communication (Sperber and Wilson, 1986), e.g., their subjective mental states like beliefs and attitudes (Kopp, 2010). Early approaches (e.g., Rich and Sidner, 1998) have tried to derive those states from the context of a shared collaborative task the agents (a user and a system) are engaged in: verbal acts in a ‘collaborative discourse’ were processed based on their linguistic structure as well as a shared plan (intentional structure) and an attentional focus (Grosz and Sidner, 1986). Heeman and Hirst (1995) presented a plan-based model for interactively solving collaborative reference in dialog. This model accounts for the generation and understanding of referring expressions and involves proposing an expression, judging and potentially clarifying it, rephrasing it, and, eventually, accepting and adopting it. However, at the same time, those plan-based attempts have been found to be too complex and brittle.

Even though, it is important to note that and how they have tried to incorporate aspects of cooperation and joint co-construction. And even up to today, almost all existing ECAs [e.g., Max (Kopp et al., 2005), Greta (Bevacqua et al., 2010), and Virtual Justina (Kenny et al., 2008)] rest on a modular architecture characterized by a multi-step processing along different routes to enable a highly dynamic and responsive dialog behavior. For example, the feedback-giving system presented by de Kok and Heylen (2012) first processes input and then determines the appropriateness of an online listener response, while “*How Was Your Day?*” prototype of Crook et al. (2012) combines a “long” loop for intent planning with a shorter loop to handle interruptions, back-channel feedback, and emotional mirroring. Yet, conversational agents typically have problems with a disfluent dialog due to barge-ins, interruptions, hesitations, or long delays. While few approaches have started to apply machine learning techniques to human dialog data, e.g., in order to identify linguistic-acoustic cues or strategies to facilitate the coordination of turn-taking (Lala et al., 2018), recent neural models in dialog systems (e.g., for dialog state tracking or response generation) often still ignore turn-taking and even more so the underlying dynamics of co-construction (cf. Skantze, 2021). Instead, current dialog systems largely focus on typed input or pre-structured question-answering and command-and-control interactions, with dedicated turn-taking cues such as wake words.

A more general computational model of grounding for task-oriented dialog was developed by Traum (1994) who proposed “discourse units” as the central building-blocks of dialog, consisting of a sequence of specific “grounding acts.” The model embodies a subjective theory of grounding that an individual agent may hold. Groundedness is estimated based on introspection of the agent’s own behavior and based on the observed behavior of the interlocutor. A number of dialog systems employed the corresponding “information-state update” model for dialog management (Larsson and Traum, 2000), which builds on simplified versions of a formal discourse theory (Poesio and Traum, 1997) to capture interactivity in discourse even on the sub-utterance level. Roque and Traum (2008) distinguished different, discrete “degrees of groundedness” in their computational model of grounding within a particularly structured dialog domain. DeVault and Stone (2006) proposed a model of common ground that accounts for uncertainty of the dialog participants but eludes the necessity to define graded shared belief in terms of probabilities: each agent maintains its own subjective (“private”) grounding status, together with an estimation of the probability of this being the objective context. Bohus and Rudnicky (2008) proposed a logic-based approach that separated the task domain model and a generic dialog engine, configured with the task model and capable of employing two strategies for resolving detected ambiguity (“misunderstandings”) and several more for non-understanding, including declaration of non-understanding, requests, re-prompts, and help messages. Yaghoubzadeh and Kopp (2017) describe a dialog management approach for users with cognitive impairments. In order to ensure that user and assistant mutually understand each other correctly, the system collects and integrates evidence of understanding (including subtle nonverbal signals) and employs a flexible grounding strategy for individual pieces of information. Overall, all of these approaches account for mentalizing and co-construction, albeit only in implicit and rudimentary ways through some form of dialog states (not explicating the agents’ subjective mental states or higher-order beliefs) and predetermined grounding strategies (not allowing for flexible and adaptive incremental updates in the dialogical interaction).

### Incremental Processing

One principle that can help to reconcile the daunting complexity of processing mental states, with the demands for fluent, real-time conversational behavior is incremental processing. Indeed, incremental processing is increasingly assumed to be a key principle for natural dialog modeling. Besides being necessary for achieving low latency and thus fluent dialogs, it provides a psychologically realistic and human-like approach to tackle the inferencing (or here better called mentalizing) required for dialog: instead of reasoning exhaustively about the most probable interpretation of an utterance or the optimal grammatical-lexical choices that maximize the probability of successful understanding, incremental processing means to come up with a “good-enough” solution in a timely manner and then being able to flexibly expand, amend, or modify it later on as needed. Technical approaches to incremental processing have been proposed for almost all language processing tasks in a conversational agent: speech recognition/synthesis, natural language understanding (Atterer et al., 2009), dialog management (Buss and Schlangen, 2011; Traum et al., 2012), and natural language generation (Skantze and Hjalmarsson, 2010; Buschmeier et al., 2012). Skantze and Schlangen (2009) proposed an abstract framework of incremental processing in dialog agents and describe a dialog system in a micro-domain that achieves incremental understanding through producing human-like clarification and grounding acts. Visser et al. (2014) describe a similarly incremental model of grounding. Hough and Schlangen (2016) present an extended version that models grounding and interactive repair in a task-oriented human-robot dialog, by tracking the dialog state in two interacting state-machines, one for its own state and one for the estimated state of its human interlocutor. Buschmeier and Kopp (2018) developed an “attentive conversational agent” that is sensitive to the vocal and non-vocal feedback of the listener, even while producing a spoken utterance itself. Based on the communicative feedback, the listener’s perception, understanding, and acceptance levels are estimated and used to adapt the agent’s communicative behavior in real-time. For example, the agent would start to produce more detailed or redundant utterances if it attributes weak understanding to the human listener. This minimal form of mentalizing, based on simple feedback signals, helped to achieve a better ration of communicative effort (time) and success. However, incrementally and collaboratively co-constructed dialogs require being able to fully reciprocate through contributions, interruptions, completions, or repairs, all flexibly and cooperatively adapted to (higher-order) representations of the mental states of the interlocutors.

## Discussion and Future Research Directions

This article aims to reflect on important issues to inform the future implementations of artificial systems that are supposed to interact with humans in spoken dialog conversation. Our main argument is that the kinds of conversational interaction we are ultimately seeking to achieve for social and collaborative agents or robots, are not likely to be attained by only trying to simulate behavioral response sequences that are typical of HHI. Rather, we will need to enable agents to cooperatively and incrementally co-construct a successful interaction with a human user. As we have argued here, the basis (and also a linchpin) for the required degree of “interaction intelligence” are coordinative mechanisms such as partner-specific adaptation of multimodal utterances, responsive turn-taking, informative feedback, or collaboratively resolving misunderstandings. As an example of the quality of cooperative interaction that we mean here, and which is fundamental and prevalent in human-human communication, is the “repair” of communication problems in dialog. It has long been pointed out how they are really treated as joint problems and hence prevented or solved collaboratively by interlocutors (Clark, 1994). All of these are hallmarks of the two primacies of communication (mentalizing and joint co-construction) that we advocate to take into account more strongly when developing interactive agents.

The current mainstream of spoken language technology predominantly follows a data-driven approach akin to “computational behaviorism,” and the two primacies of human communication, despite the overwhelming theoretical and empirical evidence for their importance, have been moving out of focus of technological approaches. This is understandable given the impressive abilities of modern deep learning techniques to extract features and patterns from large amounts of example data. However, despite the advances in fields, such as language recognition, translation, or response generation, it can be doubted that data-based models – even with the possibilities it might provide in 10 years’ time – will be able to join in the highly interactive and context-dependent processes of communication and meta-communication, which can take unforeseeable turns, respond to deeply rooted mental states, and are collaboratively shaped by two or more interaction partners. For one thing, even though dialog system models acknowledge the importance of internal states by semantic decoding or belief tracking, this most often boils down to describing the state of the conversation as a whole. Likewise, cooperativity is usually mapped out implicitly in the form of an optimized dialog policy. While this proves sufficient for pre-structured dialog services (and is nowadays employed in commercial systems), it is not an adequate model of the different mental perspectives that interactants hold as well as the process in which they incrementally establish intersubjective understanding through verbal and nonverbal communicative acts.

Another challenge of current AI-based approaches is their lack of transparency. Such a system will employ “black box” models with many parameters (175 billion in GPT-3) that have been adjusted to globally optimize for certain criteria. It will thus be hard to decipher how and why it produces a specific behavior – a problem widely acknowledged and addressed in current research on “Explainable AI” (Lim et al., 2009; Rader et al., 2018). Machine learning, thus, is a good way to foster applications, but it is still open how it can support progress in Cognitive Science and understanding the human mind and its communicative abilities. What’s more, in HAI also, the human user will always engage in reasoning about the inner (“mental”) states and abilities of the artificial interaction partner, thus facing similar problems. This is amplified by the fact that artificial entities may exhibit isolated “super-human” abilities, e.g., by accessing hidden knowledge sources or sensing signals that humans cannot perceive or will not expect the system to know.

What is the best path to choose in order to advance human-agent communication? Much current research has been and is being directed to understanding natural language input, interpreting nonverbal behavior and social signals, synthesize expressive spoken utterances, or realizing the concurrent, incremental perception and production of multimodal, multi-functional expressions. While all of these endeavors are important, we contend that they will remain solitary unless we succeed in providing artificial systems with the underlying ability to *intertwine incremental mentalizing and socio-communicative behavior* at the dialog level. As pointed out above, this becomes evident when considering the interaction problems that abound in human-agent communication, for example, misrecognitions, misinterpretations, overlaps, interruptions, or discontinuations. Such imperfections happen frequently in human-human communication too, but the human ability for incremental cooperation and joint co-construction affords robust and efficient means of dealing with them (Clark, 1994; Dingemanse et al., 2015), even to the extent that humans may not perceive them as “problems” but as normal stages in the cooperative process of jointly creating understanding.

Work in human-agent communication and conversational agents has started to address issues like error detection and recovery (Skantze, 2007; Bohus and Rudnicky, 2008). The results also point to the fact that one cannot rely on fixed error handling strategies executed by the system but has to work toward enabling flexible and *cooperative* repair processes between both human and agent. In a more recent paper, Purver et al. (2018) discuss and evaluate computational models of miscommunication phenomena, specifically for self- and other-repair detection. They likewise identify incrementality of processing and robustness to sparsity to be requirements for satisfactory models. Overall, however, only small parts of the preventive or repair strategies that humans are found to employ jointly and cooperatively in order to ensure communicative success, have been realized in artifacts. What is missing in particular are conceptual-computational approaches that combine and integrate those mechanisms grounded in a computational account of mentalizing and incremental co-construction. To that end, future research will need to make progress in different directions:

### Multi-Layer Model of Cooperative Interaction

We conjecture that a conversation between two individuals is characterized by dynamic *interpersonal coordination processes* that unfold incrementally and in different forms. We suggest to develop architectures that incorporate, at least, three different *layers of cooperation and coordination* characterized by different forms of communicative efforts and socio-cognitive processes (Allwood et al., 1992; Buschmeier and Kopp, 2018):

- *Contact*: At the lowest level, interlocutors build hypotheses about the question whether there is contact in the sense that interlocutors perceive each other and pay attention (“*are you with me?*”). This bears resemblance to concepts of rapport-building (Huang et al., 2011). Incremental co-construction and mentalizing, here, will rest on (fast) perceptual processing and fast state adjustments of relevant cues such as spatial orientation, gaze, joint attention, and properly timed and relevant feedback signals.
- *Understanding*: This layer is directed toward the perception whether co-construction of content is successful and whether the communicative intention is met (“*do you get my point?*”). Cooperation is realized by incremental adaptation according to hypotheses of whether a mutual understanding is achieved or not. This layer also includes Allwood’s perception function and is the arena of Clark’s notion of grounding of shared meaning. Here, fundamental human abilities such as increasing understanding and communicative success *via* alignment (Branigan et al., 2010) play a role.
- *Plans and goals*: Here, overarching goals of one’s own as well as of the interaction partner are considered (“*are our goals aligned?*”). This includes reasoning about the other’s plans, goals, and desires, as well as the coordination of inter-dependent intentions or plans through means of, e.g., proposing, negotiating, or adopting.

We assume a loose coupling and, at least, partial self-containment of the coordinative processes at these different layers. One example that most of us know is when talking on the phone to somebody without actually being interested in the conversation. Only by providing affirmative, yet generic feedback at the right times, thus cooperating at the Contact Layer, one can successfully create the illusion of understanding without actually extracting meaning. Modeling the layers may be done with corresponding hierarchical and factorized models with corresponding representational features (e.g., semantic representations with levels of grounding at the Understanding Layer; Roque and Traum, 2008; Buschmeier and Kopp, 2018) and dedicated policies for the respective coordinative behavior.

### Minimal or “Satisficing” Mentalizing in Dialog

Social agents need to be able to recognize mental perspectives of interactants. But how and when can an agent know what the user has perceived or understood? How can an agent know whether itself has understood the user? How can an agent know what the user assumes it has understood? Such questions are classical in mental perspective-taking and ToM research as well as in dialog theory, but we do not have adequate computational models of this in human-agent communication. For instance, at the Understanding Layer an interlocutor has to maintain and distinguish, at the very least, between beliefs concerning (1) her understanding of the interlocutor’s utterance (“*me*-*belief*”), (2) the interlocutor’s understanding of her utterance (“*you*-*belief*”), and (3) the extent and degree to which they both assume to have an intersubjectively shared understanding (“*we*-*belief*”). While this in principle can be taken ad infinitum, we conjecture that 1st-order and 2nd-order beliefs are necessary and sufficient, along with the notion of mutually shared beliefs, to explain already a broad range of dialog phenomena (e.g., using social gaze to establish mutual awareness of one’s communicative intention and the other’s attention to it). These mental processes form the socio-cognitive basis of shared intentionality in communication (cf. Tomasello, 2003), and they are likely to differ between the three layers and now come to be coupled in interaction. Crucially, mentalizing can quickly become complex and even intractable (e.g., when treated as inverse planning). Thus, it can hardly be a fixed pattern of processing but must be adaptive in itself depending on the needs of the current situation, the hypotheses the agent needs to build and test, as well as the temporal or computational resources available. For example, in the case of communication problems or conflicts, an agent needs to engage in deeper yet more costly levels of mentalizing, for which additional time will be required. That is, we need factored models for fast-but-coarse minimal mentalizing (Buschmeier and Kopp, 2018), but also for increasing the “mentalizing depth” when necessary. Current probabilistic frameworks such as Bayesian ToM and bounded rationality may provide a computational account for modeling such an adaptive mentalizing (Pöppel and Kopp, 2018), but yet have to be applied to dialog modeling.

### Prediction-Based Incremental Processing

Frith and Frith (2006) state that mere knowledge will not be enough to successfully mentalize: “*The bottom line of the idea of mentalising is that we predict what other individuals will do in a given situation from their desires, their knowledge and their beliefs, and not from the actual state of the world*” (Frith and Frith, 2006, p. 6). We agree and suggest that interactive agents, too, need to operate on the aforementioned (inter-)subjective representations to pursue goals in the form of desired internal states and to derive predictions of (1) how the interlocutor is likely to behave next, (2) what the interlocutor is likely to pursue with this behavior (i.e., wants me to understand and do), and (3) how the interlocutor will understand my own social behavior (and how I should thus behave in order to achieve my goals). They are continuously updated based on these predictive processes and the confirming or deviating evidence obtained in the ongoing interaction. For example, a speaker may form predictions and expectations of what the listener should be able to understand (you-beliefs), and may use this to tailor her utterances and to evaluate subsequent listener responses. How these processes play out in social behavior, different modalities, or at different points of a communicative interaction still needs to be understood and modeled computationally. Particularly important questions relate to how low-level perceptual processing interacts with mentalizing, and how predictive processes come to interact and couple with each other across interactants (Kahl and Kopp, 2015).

**Figure 2:**
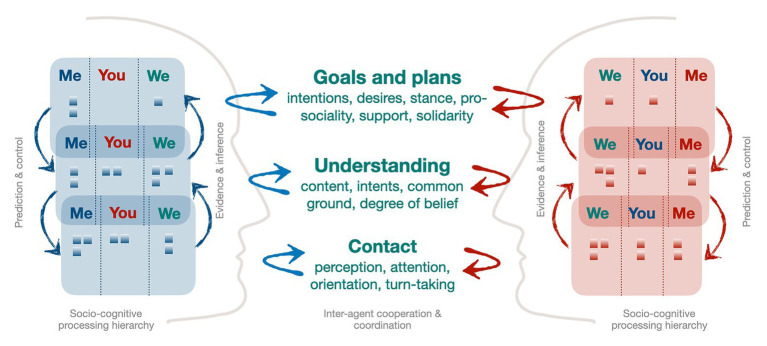
A multi-layer model of cooperative human-agent interaction based on mentalizing and incremental, joint co-construction.

Figure 2 provides a symbolic and coarse illustration of the basic principles according to which we envision successful models of HAI to be structured (expanding Figure 1). It shows the basic levels of inter-agent coordination (see above) along with the corresponding aspects being coordinated at each layer. Each agent forms and maintains relevant intentions and beliefs (symbolized as the little boxes) for three different (inter-)subjective mental perspectives. These mental states are processed *via* hierarchical prediction, control, and evidence-based inference mechanisms. In addition, and not shown here, each agent is expected to be able to map mental states to/from communicative behavior at the respective layer and using appropriate semiotic systems (e.g., by means of language understanding and generation). This model extends a previous proposal on embodied coordination (Kopp, 2010) to a more comprehensive conceptual framework for building agents capable of the cooperative mechanisms of conversation. Note that we, here, aim to make the basic arguments for this kind of general model and overall concept, the detailed formulation and implementation of which pose a long-term research program.

To conclude, conversational agents that are living up to the primacies of mentalizing and incremental joint co-construction can only be expected to come into existence very much in the future, and they require substantial and collaborative research in Cognitive Science, Psychology, and Computer Science. We have suggested to focus on those crucial abilities that underlie human communication. Working toward them will require an integrative effort. For one thing, it will certainly require (and possibly inspire) modern ML-based techniques to deal with the high-dimensional, non-linear mappings between sensory data, feature-based representations, and action policies in communicative interactions. What we advocate here is integrating these methods with model-based approaches to realize the aforementioned socio-cognitive and behavioral processes operating on the hidden states that cannot be observed in data. It will also require the close integration of modeling and evaluation through experimental studies on the effects of and problems within human-agent communication, to analyze whether systems that have first basic abilities to mentalize and incrementally co-construct are actually better communicators. While all of these ideas need to be detailed out in further research, we think that it is important to re-instantiate them as important research goals and to embrace them as a basic mindset when developing future interactive agent systems.

## Author Contributions

All authors listed have made a substantial, direct and intellectual contribution to the work, and approved it for publication.

### Conflict of Interest

The authors declare that the research was conducted in the absence of any commercial or financial relationships that could be construed as a potential conflict of interest.

## Footnotes

^1^https://chrisgpotts.medium.com/is-it-possible-for-language-models-to-achieve-language-understanding-81df45082ee2

^2^Yann LeCunn (https://www.facebook.com/yann.lecun/posts/10157253205637143).
